# Effect of Metabolic and Bariatric Surgery Associated with Changes in Weight Loss, Free Leptin Index, and Soluble Leptin Receptor

**DOI:** 10.3390/metabo15120764

**Published:** 2025-11-25

**Authors:** Lourdes Basurto, Norma Basurto-Acevedo, Norma Oviedo, Erika Santa Cruz-Galicia, Ana Isabel Rodríguez-Martínez, Emiliano Tesoro-Cruz, Carlos Martínez-Murillo, Ariana Grisel García-Estrada, Andrea Cristina Nájera Meneses, Leticia Manuel-Apolinar

**Affiliations:** 1 Investigación, Hospital de la Mujer, CCINSHAE, Secretaría de Salud, CCINSHAE (Comisión Coordinadora de los Institutos Nacionales de Salud y Hospitales de Alta Especialidad), Mexico City 11400, Mexico; maria.basurto@salud.gob.mx; 2 Servicio de Cirugía General, Clínica de Tracto Digestivo Superior, Cirugía Bariátrica y Metabólica, Hospital General de Mexico Dr. Eduardo Liceaga, Hospital Ángeles Metropolitano, Mexico City 06720, Mexico; normabasurtoa@outlook.com; 3 Unidad de Investigación Biomédica en Infectología e Inmunología, Hospital de Infectología, Centro Médico Nacional “La Raza”, IMSS, Col. La Raza, Mexico City 02990, Mexico; norma.oviedo@imss.gob.mx (N.O.); emiliano.tesoro@imss.gob.mx (E.T.-C.); 4 Unidad de Investigación Médica en Enfermedades Endocrinas, Centro Médico Nacional Siglo XXI, Instituto Mexicano del Seguro Social (IMSS), Mexico City 06720, Mexico; galiciaerika536@gmail.com (E.S.C.-G.); mc20roma2078@facmed.unam.mx (A.I.R.-M.); 5 Servicio de Hematología, Hospital General de Mexico Dr. Eduardo Liceaga, Secretaría Salud, Mexico City 06720, Mexico; investigacionhm@salud.gob.mx; 6 Unidad de Investigación Médica en Enfermedades Endocrinas, Hospital de Especialidades, Centro Médico Nacional Siglo XXI, Instituto Mexicano del Seguro Social, Mexico City 06720, Mexico; griselge28@gmail.com (A.G.G.-E.); andynajme@gmail.com (A.C.N.M.)

**Keywords:** bariatric surgery, percentage of excess weight loss, soluble leptin receptor, leptin, ghrelin

## Abstract

**Objective:** To compare the clinical and metabolic impact of the relationship between leptin, serum soluble leptin receptor (sOB-R), free leptin index (FLI), and ghrelin among different metabolic and bariatric surgeries (MBSs) in patients with severe obesity. **Method:** Cohort study including 194 patients >18 years old diagnosed with obesity (body mass index (BMI) ≥ 30 kg/m^2^) undergoing bariatric surgery at the General Hospital of Mexico. Participants were distributed into three surgical groups: Roux-en-Y gastric bypass (RYGB) (n = 36), one anastomosis gastric bypass (OAGB) (n = 86), and sleeve gastrectomy (SG) (n = 72); all operations were performed laparoscopically. Pre- and post-surgical measurements were taken, including anthropometric measurements, lipid profile, glycated hemoglobin (HbA1c), leptin, sOB-R, ghrelin, and FLI. Protocol registration: DI/16/304/04/090. **Results:** A total of 194 patients with obesity were analyzed, the average weight was 114.9 ± 24 kg, and the preoperative BMI was 43.1 ± 8.0 kg/m^2^. Three types of MBSs were compared. Post-surgery, all groups showed a significant percentage of excess weight loss (%EWL), for example, in OAGB 65.6 ± 12.2%EWL at 12 months (*p* < 0.0001). In addition, ghrelin levels decreased significantly, especially in a short time compared with pre-surgery levels (from 4 ± 2.5 to 1.6 ± 1 ng/mL per first week (*p* < 0.0001)). Similarly, leptin diminished in a short time (*p* < 0.01). Soluble leptin receptor showed differences in the biochemical behavior of leptin, with FLI decreasing significantly (*p* < 0.003) after one year (*p* = 0.001). **Conclusions:** All techniques were effective in reducing body weight, %EWL, and hormonal modulation during the first three months. In addition, ghrelin and FLI levels partially increased as a physiological adaptation to weight loss and resumption of food intake, without reaching initial values.

## 1. Introduction

During the last five decades, the incidence of obesity has surged dramatically, reaching levels comparable to a global pandemic. Currently, the number of adults classified as overweight exceeds one billion worldwide, with at least 300 million individuals meeting the criteria for clinical obesity [1]. Thus, obesity is one of the most urgent health problems in modern times because of its epidemic prevalence, high disease burden, and high mortality [1,2]. Defined by the excessive growth of white adipose tissue (WAT), obesity contributes to a variety of health issues and is a significant risk factor for chronic illnesses, including cancer, cardiovascular disease (CVD), and diabetes mellitus (DM). A percentage of morbid or extreme obesity requires metabolic and bariatric surgery (MBS). This method, alongside the best medical therapy available, results in greater weight reduction than the use of both lifestyle modifications and pharmaceutical interventions [2]. Therefore, MBS modifies adipokines and metabolic markers.

Adipose tissue is a loose connective tissue; it is a bioactive organ mainly consisting of adipocytes. This tissue stores energy in the form of lipids and plays a major endocrine role by secreting several soluble factors known as adipokines [3]. Thus, leptin is an adipokine produced by the adipose tissue. This protein, which weighs 16 kDa and is encoded by the *ob* gene, plays a role in body weight regulation by decreasing the desire to eat through central regulation and increasing energy expenditure [4,5,6], and it regulates metabolic activities in peripheral tissues and affects both reproductive functions and immune system responses [7,8]. In addition, it is involved in obesity with insulin resistance (IR) and cardiovascular disease, partly via direct action on pancreatic β-cells and hepatocytes [8]. Some studies report that fat distribution such as visceral adipose tissue (VAT) is strongly associated with proinflammatory signals and proatherogenic cytokines of adipocyte. Thus, excess body fat is a leading factor in both morbidity and mortality largely due to its influence on lipid profiles in the bloodstream [8,9,10].

Leptin is often associated with the observed correlation between overall body fat and the concentration of leptin generated by adipose tissue [3,11]. Both the amount of leptin produced and the expression of membrane-anchored leptin receptors, which transmit signals, play roles in this process. Leptin exerts its effects on target tissues by binding to membrane-bound receptors that are widely distributed throughout the body, leptin-binding proteins modulate the efficacy of leptin signaling and influence the leptin sensitivity of various tissues [10,11,12]. As a result of the cleavage process, the soluble leptin receptor is generated, which serves as the primary leptin-binding protein in human circulation and regulates its bioavailability [13]. The sOB-R belongs to the class I cytokine receptor family and is characterized by a single transmembrane domain. The levels of sOB-R vary depending on the metabolic condition, including disorders like type 1 diabetes mellitus and obesity, which can lead to either increased or decreased leptin sensitivity [13].

Leptin interacts with soluble leptin receptor, and the ratio between circulating leptin and sOB-R—known as the free leptin index—serves as an indicator of leptin activity. While leptin levels are positively associated with obesity and insulin resistance, it remains unclear whether sOB-R and FLI show similar correlations [13,14,15]. Consequently, sOB-R plays a regulatory role in determining the availability of leptin and its downstream cellular actions [14,15].

On the other hand, ghrelin is an orexigenic hormone that also acts on adipose tissue. Recent studies have related that this hormone favors the accumulation of lipids in visceral fat. Specifically, it causes overexpression of fat genes involved in lipid retention [3]. The fat accumulated in the abdomen region is considered the most harmful, since it leads to the appearance of comorbidities [16,17]. In addition, it has been discovered through in vitro studies that ghrelin influences the inflammatory process and angiogenesis through alterations of its GHSR1a receptor [18,19]. It is unknown how ghrelin, sOB-R, FLI, and leptin are modified after MBS.

The purpose of our investigation was therefore to study the courses of sOB-R, FLI, leptin, and ghrelin serum concentrations associated with patterns of change in extremely obese patients after MBS, and to link them to clinical parameters, such as BMI, glucose, and lipid metabolism.

## 2. Materials and Methods

### 2.1. Ethics Approval

This was a prospective, nonrandomized cohort study involving patients who underwent metabolic and obesity surgery. This study was carried out according to the World Medical Association (WMA) Declaration of Helsinki (2000) and the Protocol to the Convention on Human Rights and Biomedicine (1999). In addition, the study procedures conformed to ethical principles and to the General Health Law for Health Research in Mexico (NOM-008-SSA3-2017) [20]. A Local Ethics Committee approved the study protocol with registered number DI/16/304/04/090 from the General Hospital of Mexico. All the study participants provided informed consent before they were included in the trial.

### 2.2. Study Population

This is a cohort study on biological samples (peripheral venous blood; serum or plasma) from subjects with morbid obesity before and after metabolic and bariatric surgery. For all individuals, the following data were obtained: affiliation, personal history, anthropometric data, and presence of comorbidities.

Participants were considered eligible for inclusion if they met the following criteria: (i) 18–60 years old, (ii) a diagnosis of obesity (BMI ≥ 30) with an indication for bariatric surgery, and (iii) failure of prior drug treatment. Exclusion criteria encompassed patients with mental incapacity, those unwilling to consent or unable to understand the study due to language barriers, pregnant or breastfeeding women, women planning pregnancy or not using adequate contraception, individuals with severe chronic inflammatory or systemic diseases, active substance abuse (alcoholism, drug addiction); uncontrolled hypothyroidism or coagulation disorders, and extreme surgical risk due to advanced cardiopulmonary diseases. Additionally, patients with any condition that, in the opinion of the investigator or treating physician, could interfere with the study or compromise the participant’s safety were excluded from participation.

They were studied at the General Hospital of Mexico. Initially, patients who were included in the protocol were oriented and educated on the context of their disease and the consequences and possible treatments, clearly and concisely specifying the characteristics of bariatric surgery.

Patients were prepared for surgery through a comprehensive evaluation, including a detailed medical history and lifestyle assessment. Laboratory investigations included complete blood count, liver function tests, erythrocyte sedimentation rate (ESR), renal function tests, lipid profile, HbA1c, and blood glucose measurements. Patients were instructed to follow a four-week dietary regimen consisting of low-fat and low-carbohydrate diets to reduce liver volume and facilitate surgical procedures.

### 2.3. Study Protocols for Metabolic and Bariatric Surgery

Relevant anthropometric measurements—such as age (years), height (cm), weight (kg), body mass index (BMI, kg/m^2^), and waist circumference (cm)—were obtained during a standardized clinical evaluation. Body composition was assessed utilizing bioelectrical impedance (Body Composition; Tanita TBF-215. Tokyo, Japan) following standardized procedures. As part of the clinical assessment, measurements were taken for waist circumference, blood pressure, and routine biochemical parameters.

All surgical interventions were conducted laparoscopically by a consistent surgical team. No significant intraoperative complications occurred, nor was there any need to convert to open laparotomy. The surgical procedures performed on participating patients were (a) sleeve gastrectomy (SG); (b) Roux-en-Y gastric bypass (RYGB); and (c) one anastomosis gastric bypass (OAGB).

Post-surgical assessment was performed in the Clinic of Obesity and Surgery Bariatric of the General Hospital of Mexico. Postsurgical dietary adaptation was a structured, dynamic, and personalized process supervised by the multidisciplinary team. It included 5 stages. In phase 1, on days 1–2 after the operation, the patients received a diet with clear liquid. In phase 2, from day 3 to week 2, a complete liquefied diet with protein and fat-soluble vitamins was used. In phase 3, from weeks 3–5, a puree-like diet with a semi-solid texture was used. In phase 4, from weeks 6 and 7, a semi-solid diet was used. In phase 5, starting from week 8, a progressive solid diet was used, with multivitamin supplementation, including cyanocobalamin and folate, iron, calcium, and vitamin D. Participants were evaluated in an outpatient clinic at 1 week and at months 1, 3, 6, and 12.

Also, during this study, we registered the mean BMI (pre-surgery, before revision, and after surgery) and average percentage of excess weight lost (%EWL) at the 12-month follow-up [21].

### 2.4. Sample Preparation and Biochemical Assay

Serum and plasma samples were obtained from a clinical study designed to investigate leptin, its soluble receptor (sOB-R), and ghrelin. Samples were collected in tubes with or without anticoagulant and centrifuged at 640× *g* for 15 min, and serum aliquots were isolated and prepared for analysis. Serum concentrations of glucose (mg/dL), total cholesterol (TC) (mg/dL), high-density lipoprotein cholesterol (HDL-C) (mg/dL), and triglycerides (TG) (mg/dL) were quantified using a Spin 120 analyzer with a colorimetric assay (Spin SX, Barcelona, Spain). Low-density lipoprotein cholesterol (LDL-C) concentrations were calculated using the Friedewald formula. Glycated hemoglobin (HbA1c) was measured using a Technicon RA1000 analyzer (Bayer Diagnostics (Leverkusen, Germany)).

### 2.5. Assay of Ghrelin, Leptin, Soluble Leptin Receptor Levels, and Free Leptin Index (FLI)

Human total ghrelin was assayed in plasma by a highly specific sandwich enzyme-linked immunosorbent assay (ELISA) method (Human Ghrelin Platinum eBioscience, BMS2192, Thermo Fisher Scientific Inc., Vienna, Austria); concentrations were ng/mL.

Plasma concentrations of leptin and sOB-R were determined following the manufacturer’s instructions using ELISA kits. Leptin levels were quantified with the Human Leptin DuoSet ELISA (Catalog No. DY398-05, R&D Systems, Inc., Minneapolis, MN, USA) and sOB-R levels with the Human Leptin Receptor DuoSet ELISA (Catalog No. DY389, R&D Systems, Inc., Minneapolis, MN, USA). The detection limits for leptin and sOB-R were 0.16 ng/mL and 0.057 ng/mL, respectively. The intra- and inter-assay coefficients of variation were below 8% for ghrelin, 6.7% for sOB-R, and 4.9% for leptin. All concentrations are expressed in ng/mL. The FLI was calculated using the following formula: FLI = leptin concentration (ng/mL)/sOB-R concentration (ng/mL).

### 2.6. Statistical Analyses

Categorical variables were presented as frequencies and percentages. Quantitative data were first evaluated for normality using the Kolmogorov–Smirnov test. Depending on the data distribution, results were expressed as mean ± standard deviation (SD), as standard error of the mean (SEM), or as median and interquartile range (IQR), as appropriate. Associations between laboratory parameters were examined using Pearson’s correlation analysis. Multiple linear regression models were applied to identify factors independently associated with leptin, sOB-R, BMI, triglycerides (TG), and ghrelin levels, after adjustment for potential confounding variables. A two-tailed *p*-value < 0.05 was considered statistically significant. All analyses were performed using GraphPad Prism software (version 10.1; GraphPad Software (San Diego, CA, USA)).

For this study, in the case of sample size calculation, it is described in detail based on the main objective of this study and effect size before and after the study, with α = 0.05, β = 0.2, power = 0.80, SD(Δ) = 1.15, and effect size E = 0.561, indicating a number of subjects per group of N = 35.

## 3. Results

### 3.1. Clinical Characteristics and Anthropometric of Patients

**Figure 1:**
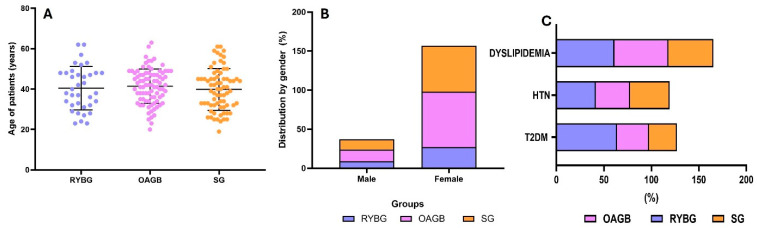
Characteristics of patients of three bariatric surgery procedures. (**A**) Age of patients by type of surgical procedure; (**B**) percentage distribution by gender in the different surgical groups; (**C**) percentage of comorbidities by surgical group.

One hundred ninety-four participants with severe obesity were enrolled, screened, and selected for metabolic and bariatric surgery. The clinical characteristics of the total study population (mean age, weight pre-surgery, comorbidity percentage of diabetes and BMI) are shown in Table 1. The group comprised 157 women and 37 men, with a mean age of 40.6 ± 9 years. The patients had a mean BMI of 43.1 Kg/m^2^ and multiple comorbidities (37% T2DM, 39% hypertension, 54% dyslipidemia, and 16% hypothyroidism). None of the subjects had severe cardiopulmonary comorbidities; only two participants had asthma controlled without systemic medications (Figure 1).

**Figure 2:**
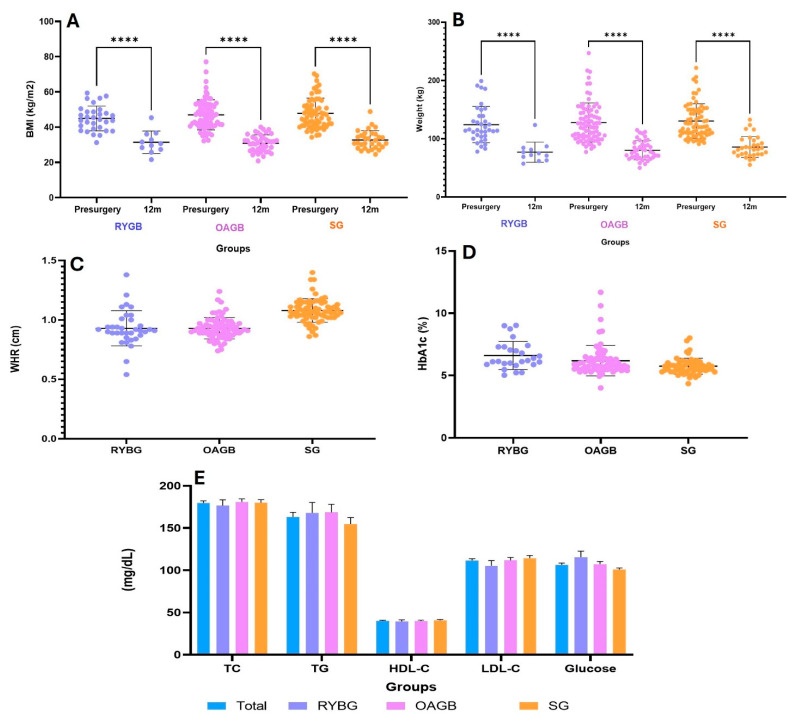
Presentation of anthropometric and biochemical variables: (**A**) BMI before surgery and 12 months after surgery by group; (**B**) pre-surgery weight and weight 12 months post-surgery by group; (**C**) WHR by surgical group; (**D**) HbA1c levels by type of MBS; (**E**) comparison of biochemical parameters between surgical groups. Data are expressed as mean ± standard deviation. (**A**,**B**). The three surgical groups showed a significant reduction in body weight 12 months post-surgery (**** *p* < 0.0001).

Also, of the MBS groups, the OAGB group had the highest basal body weight at 117.2 ± 26.4; WHR showed a tendency to increase in the RYBG group without significant difference. Of the metabolic variables, glucose had a significant difference in the RYBG vs. SG groups, * *p* < 0.05; HbA1c, *** *p* < 0.001, RYBG vs. SG (Figure 2).

### 3.2. Effect of Metabolic and Bariatric Surgery on %EWL and Metabolic Markers

Three MBS procedures were evaluated, RYGB, OAGB, and SG, which were proven to be effective in reducing body weight and improving metabolic parameters in patients with severe obesity.

The RYGB group consisted of patients with a higher metabolic burden, reflected in a higher prevalence of T2DM (63.8%), hypertension (41.6%), and dyslipidemia (61.0%), as well as higher levels of HbA1c (6.7 ± 1.0%) and glucose (115.6 ± 31.2 mg/dL) (Table 1). In comparison, patients in the OAGB group had a higher initial weight (117.2 ± 26.4 kg) and higher BMI (44.7 ± 7.8 kg/m^2^) but a lower prevalence of metabolic comorbidities (T2DM: 33.7%, HbA1c: 6.2 ± 1.3%, glucose: 107.3 ± 28.8 mg/dL). For their part, SG patients had an initial weight of 114.3 ± 20.8 kg, a BMI of 42.3 ± 8.9 kg/m^2^, and the lowest metabolic burden (T2DM: 29.1%, HbA1c: 5.6 ± 0.5%, glucose: 100.9 ± 15.9 mg/dL) (Figure 2).

**Figure 3:**
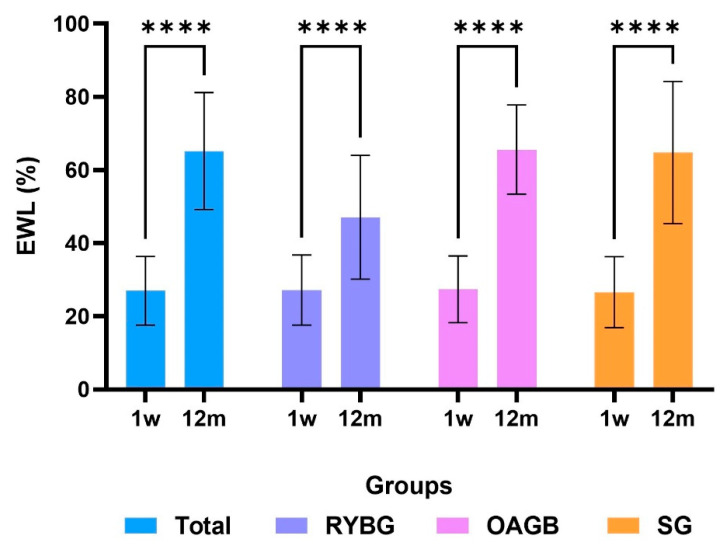
Comparison of %EWL between week 1 and 12 months post-surgery. A statistically significant difference was observed in all groups between week 1 and 12 months post-surgery (**** *p* < 0.0001). Groups were compared using a two-tailed *t*-test.

After 12 months of follow-up, significant weight loss was observed in all groups (Table 2). The OAGB group showed the greatest reduction in excess weight (%EWL) at 65.6 ± 12.2%, followed closely by the SG group at 64.8 ± 19.4%, while the RYGB group had a lower %EWL of 47.1 ± 16.6% (Figure 1). In terms of absolute weight at 12 months, the RYGB and OAGB groups achieved similar values (74.8 ± 8.6 kg and 74.1 ± 19.4 kg, respectively), while the sleeve gastrectomy group had a slightly higher final weight (83.9 ± 16.6 kg) (Table 2, Figure 3).

Other observations were that in all participants, at 12 months post-surgery, T2DM had decreased to 21.6%, HTN to 19.1%, and dyslipidemia to 16.5%. Compared to baseline levels, in the RYBG group, glucose concentration decreased significantly to 107.3 ± 34.9 mg/dL (*p* < 0.05); in the OABG group to 101.4 ± 15.3 mg/dL (*p* < 0.01); and in the SG group to 95.5 ± 13.1 mg/dL (*p* < 0.005). Triglycerides also showed a significant reduction to 140.0 ± 32.7 mg/dL (*p* < 0.05), 141.7 ± 43.5 mg/dL (*p* < 0.05), and 129.6 ± 24.7 mg/dL (*p* < 0.001) in the RYBG, OABG, and SG groups; while LDL-C decreased to 101.6 ± 27.7 mg/dL (NS), 105.9 ± 15.5 (*p* < 0.05), and 97.6 ± 38.0 mg/dL (*p* < 0.05), respectively.

Despite the lowest percentage having been observed in the RYGB group, this procedure could have metabolic advantages, given that it was applied to patients with a higher degree of glycemic dysfunction and comorbidities. In contrast, OAGB and SG proved to be more effective procedures for weight loss in patients with a lower baseline metabolic profile.

Thus, after 12 months post-surgery, fat, muscle, and water mass decreased (*p* < 0.001), respectively. Also, there were mean decreases in glucose, lipid, and HbA1c levels (*p* < 0.001).

### 3.3. Association Between Ghrelin, FLI, sOB-R, and Metabolic Variables

Serum levels of ghrelin pre-surgery were 4 ± 2.5 ng/mL, but in a short time, post-surgery ghrelin levels significantly decreased; at 1 week and 1 month, they were 1.6 ± 1.8 ng/mL and 1.7 ± 1.8 ng/mL (*p* < 0.0001), being 60% less than the levels at baseline. But at 6 m and 12 m, the levels had increased to 3.1 ± 2.4 ng/mL (Table 3).

The analysis demonstrated that the level of leptin pre-surgery was 25.9 ± 4 ng/mL; leptin decreased until 12 months post-surgery to 19.45 ± 3.4 ng/mL (*p* < 0.0001), which is different in comparison to baseline. In this case, pre-surgery sOB-R was 4.6 ng/mL and consequently pre-surgery FLI was 8 ± 3.9, which decrease over time, and at 12 months it was 5.1 (*p* < 0.0001), and it was without plasma sOB-R and leptin levels of the participants (Table 3).

**Figure 4:**
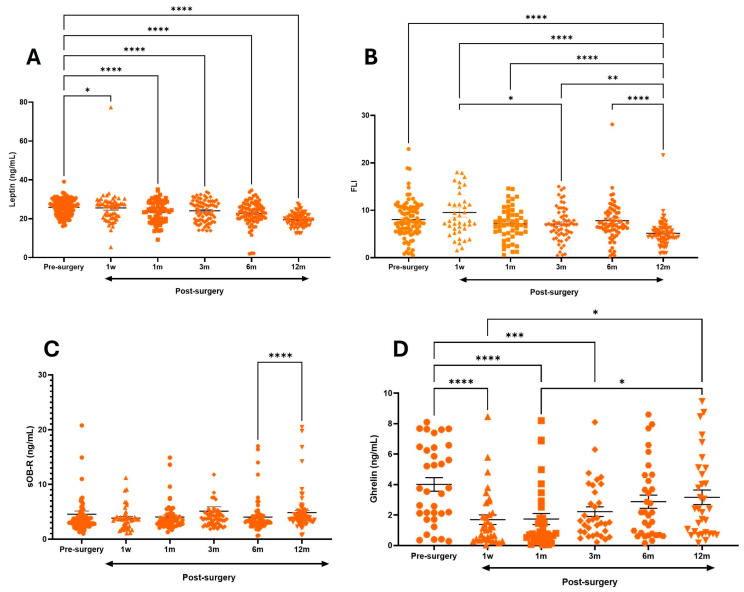
Comparison of circulating leptin (**A**), FLI (**B**), sOB-R (**C**) and ghrelin (**D**) pre-surgery and post-surgery (week 1 until 12 months post-surgery). Statistically significant differences were observed in groups post-surgery, * *p* < 0.01; ** *p* < 0.001; *** *p* < 0.005; **** *p* < 0.0001.

The present study clearly demonstrated that leptin and ghrelin decreased in the short time of one week; they were associated with sOB-R levels (*p* < 0.01) (Figure 4). The decrease in ghrelin and leptin constantly showed a tendency to decrease (*p* < 0.0001).

**Figure 5:**
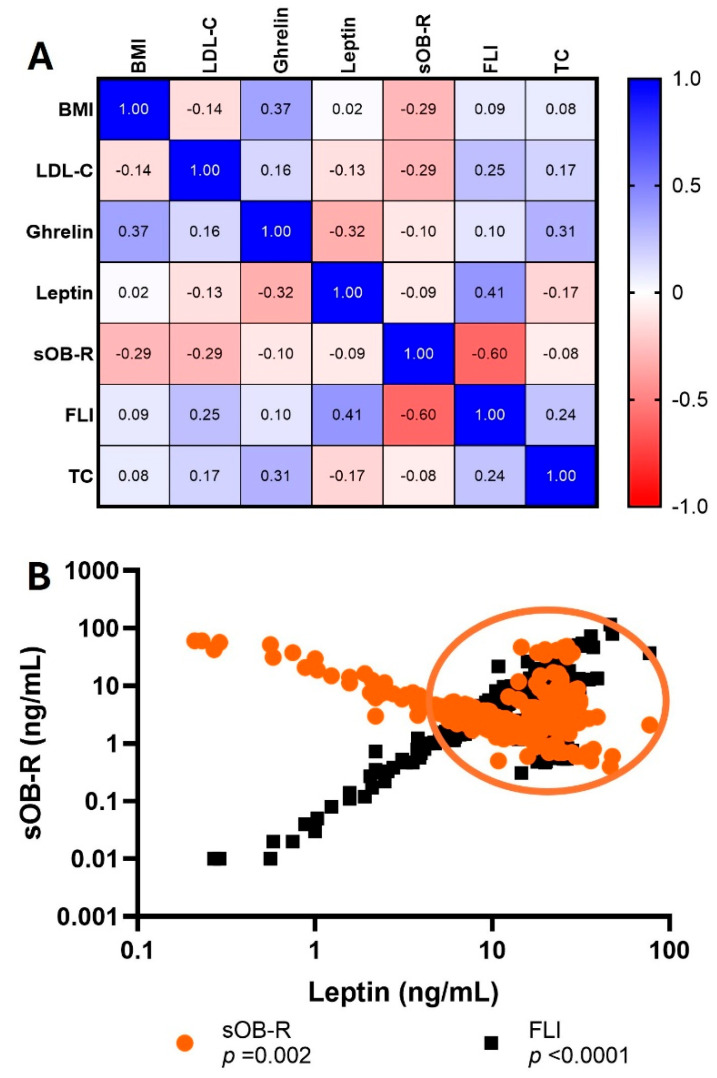
(**A**) Correlation matrix between circulating leptin, ghrelin, FLI, and anthropometric/metabolic variable changes following metabolic and bariatric surgery. Values are repeated-measures correlations (also called intra-individual correlations). Only significant results are shown based on Bonferroni-adjusted *p*-value. (**B**) Graph of correlations of sOB-R and FLI with leptin.

Figure 5 shows the correlation matrix of variables BMI, ghrelin, leptin, and sOB-R, finding a correlation of BMI vs. sOB-R (*p* < 0.02), BMI vs. ghrelin (*p* < 0.04), BMI vs. FLI (*p* < 0.02), FLI vs. leptin (*p* < 0.003), sOB-R vs. FLI (*p* < 0.001), and TC vs. FLI (*p* < 0.06).

## 4. Discussion

This study provides relevant evidence on the relationship between gastric and adipose tissue dysfunction in severe obesity and its impact on the ghrelin–leptin hormonal axis, particularly after different types of MBS. The findings confirm that the type of surgical intervention significantly influences the levels of these hormones and their relationship with metabolic parameters such as BMI, %EWL, and insulin sensitivity [21,22,23].

Severe obesity is characterized by abnormal expansion of WAT, which acts as an endocrine organ capable of secreting various adipokines, including leptin. However, the physiological action of this hormone is modulated by the concentration of sOB-R, which regulates its bioavailability through the FLI, thus conditioning tissue sensitivity [24]. On the other hand, ghrelin, a hormone with orexigenic properties primarily synthesized in the gastric fundus, is essential for the regulation of appetite and maintenance of energy balance. Under normal physiological conditions, the balance between ghrelin and leptin is essential for metabolic control. However, in the context of obesity, this balance is profoundly altered. Despite advances, it is still not entirely clear how these mechanisms behave after surgical interventions such as bariatric surgery [22,23,24].

Numerous studies have shown that, following metabolic and bariatric surgery, both WAT and VAT undergo progressive reduction throughout the weight loss process. Among the most effective surgical procedures for achieving sustained weight loss and significant improvement in metabolic comorbidities are SG, RYGB, and biliopancreatic diversion with duodenal switch (BPD-DS). In particular, scientific evidence strongly supports metabolic and bariatric surgery as an effective therapeutic option for managing type 2 diabetes mellitus, often demonstrating superior outcomes compared with conventional non-surgical medical treatments [25,26]. In line with these findings, the Swedish Obesity Study showed that a shorter duration of diabetes prior to surgery is linked to increased remission rates at 10 and 15 years post-surgery. Additionally, a 53% reduction in cardiovascular mortality was observed in patients with severe obesity who received bariatric surgery in comparison with those who did not undergo surgical intervention [27,28].

In line with this background, our results showed that the group undergoing RYGB had the highest burden of metabolic comorbidities (type 2 diabetes: 63.8%; hypertension: 41.6%; and dyslipidemia: 61.1%), suggesting a more impaired metabolic profile from the outset. This greater dysfunction could explain, at least in part, the lower %EWL observed in this group after one year compared to patients who underwent OAGB or sleeve gastrectomy [22,23,29].

Severe obesity is linked to a persistent, low-grade inflammatory state, largely due to impaired adipose tissue function. This condition manifests itself through increased expression of proinflammatory genes, immune cell infiltration, alterations in adipocyte differentiation, and reduced expression of genes involved in lipogenesis. In addition, hypertrophic adipocytes exhibit increased basal lipolysis, which promotes excessive release of free fatty acids, thereby contributing to metabolic deterioration [30,31]. It should be noted that there are functional and developmental differences between WAT and VAT, even in adulthood, which conditions different physiological responses to metabolic interventions [32]. Added to this is the fact that adiposity is a polygenic trait regulated by multiple genes and molecular pathways, which affect both its phenotypic expression and the individual response to treatments [22].

In this scenario, the type of MBS can play a key role in restoring hormonal balance, especially with regard to the ghrelin–leptin axis, which is fundamental in regulating appetite and energy metabolism. For example, RYGB, by combining restrictive and malabsorptive mechanisms, has been shown to significantly reduce leptin levels and alter ghrelin levels, thereby promoting an improvement in the metabolic profile. Similarly, OAGB, a simpler technique with a single anastomosis, achieves notable reductions in leptin and some stabilization of ghrelin levels; however, high preoperative values of the latter could correlate with lower weight loss efficacy. On the other hand, SG, being a purely restrictive procedure involving resection of the gastric fundus (the main site of ghrelin production), produces a significant decrease in both hormones [22,23].

The results obtained in this study confirmed that patients with severe obesity had a hormonal profile characterized by hyperleptinemia and hyperghrelinemia, conditions associated with systemic metabolic dysfunction. However, after surgery, the concentrations of both hormones markedly declined during the first postoperative month. It is important to note that, although this reduction was sustained in the case of leptin, ghrelin levels showed a partial recovery at 6 and 12 months, especially in surgical procedures with a malabsorptive component. At the same time, there was a substantial improvement in clinical parameters such as BMI, %EWL, and body fat mass.

Experimental studies in rats have demonstrated that ghrelin promotes preadipocyte differentiation and adipogenesis, suppresses adipocyte apoptosis, and counteracts lipolysis [33]. It has also been shown that ghrelin contributes to body weight gain by increasing adiposity independently of food intake [34]. Several reports indicate that both gastric ghrelin cell density and circulating ghrelin levels are higher in females than in males, suggesting that ghrelin secretion may be influenced by sex hormones [35]. In line with this, experiments using isolated stomach cells have revealed that estrogen treatment significantly elevates ghrelin mRNA expression and the number of ghrelin-immunoreactive cells [36], and other studies have further confirmed that estrogen enhances circulating ghrelin levels [37].

Moreover, reductions in body weight following metabolic and bariatric surgery have been associated with decreased ghrelin concentrations [35]. Recent evidence also indicates that melanocortin 2 receptor accessory protein 2 (MRAP2) modulates the ghrelin receptor GHSR1a by suppressing its constitutive activity, enhancing its G protein-dependent signaling, and preventing β-arrestin recruitment and signaling in response to ghrelin [36,38], suggesting potential therapeutic implications for obesity. Ghrelin plays a key role in regulating energy homeostasis, stimulating appetite and promoting fat storage in the short term and increasing overall body weight and adiposity in the long term [39]. During fasting, ghrelin levels rise, stimulating food intake and reducing energy expenditure, while favoring carbohydrate utilization and promoting glucose release from hepatocytes [37].

In the case of leptin–ghrelin, there was deregulation of these hormones under conditions of increased adipose tissue that was combined with metabolic alterations in severe obesity. Thus, as it is known that sOB-R acts to regulate bioavailability of leptin, we suggest future studies to clarify the molecular mechanisms underlying ghrelin’s effects, as well as sOB-R’s effect on obesity (Figure 5), since it was observed that, in hyperleptinemia and hyperghrelinemia, the sOB-R modifies this response. Thus, it could be a modulator of balance of ghrelin–leptin with inflammation or insulin sensibility [24]. In this study, a particularly relevant finding was the change in sOB-R behavior after surgery. Its decrease following surgical treatment appeared to be partially influenced by ghrelin dynamics, which could have important implications for the improvement in insulin resistance observed in some patients. Along these lines, Hahn et al. proposed that a reduction in sOB-R levels could constitute a transient compensatory mechanism to counteract leptin resistance, allowing for a partial restoration of the functionality of this hormone in obese individuals [40].

In the context of obesity, it has been shown that elevated leptin levels reflect a dysfunction in its physiological action, which translates into a state of leptin resistance. Several mechanisms have been proposed to explain this phenomenon, including alterations in intracellular receptor signaling, impairments in leptin transport across the blood–brain barrier, and dysfunctions in the structure or expression of the receptor itself. Also, multiple mutations in the leptin receptor have been identified in patients presenting with early-onset severe obesity and hyperphagic behavior [41]. The results of this study support this hypothesis, as it was observed that obese patients had elevated leptin concentrations accompanied by a reduction in sOB-R levels. This combination led to an increase in the FLI in the first week, which could be interpreted as an indirect marker of persistent leptin resistance.

On the other hand, the results obtained suggest that satiety and appetite regulation could be modulated by different physiological mechanisms depending on the type of bariatric surgery performed. Specifically, it was observed that sleeve gastrectomy did not appear to induce significant improvements in leptin sensitivity, unlike RYGB, which is consistent with previous studies which also did not report a clear effect of SG on leptin sensitivity. These differences between the two surgical procedures could have relevant clinical implications, as they show that not all bariatric techniques have the same impact on appetite modulation and metabolism [29]. Therefore, further research is needed to elucidate the specific physiological mechanisms associated with each intervention.

Although our study demonstrated significant hormonal and metabolic improvements after bariatric surgery, Morante et al. reported that moderate weight loss induced by dietary intervention decreased leptin and increased ghrelin concentrations yet did not significantly alter subjective appetite sensations, indicating that hormonal counter-regulation may hinder sustained weight reduction. Nevertheless, these adaptations appear less pronounced than those observed after bariatric procedures [42].

The role of leptin and its receptor sOB-R go beyond its known function in the immune response to infections. In fact, this hormone has been implicated in a wide range of chronic diseases, including cardiovascular disease, neurological disorders, insulin resistance, osteoporosis, and even certain types of cancer. These associations are supported by studies that show a close relationship between leptin levels and an individual’s metabolic and immune status [43]. Understanding the role of leptin in the host immune response and in the progression of various diseases during voluntary or surgically induced weight loss processes represents a crucial challenge for future research. In particular, it is interesting to explore the possible structural and evolutionary relationship between leptin, its receptor, and other cytokines such as G-CSF and IL-6, along with their specific receptors. This similarity has allowed these systems to be used as models to further study the leptin–leptin receptor (leptin-sOB-R) axis.

In this context, another relevant issue is determining whether the use of drugs modifying cardiovascular risk factors, such as antihypertensive, antihyperlipidemic, and antihyperglycemic drugs, can influence plasma leptin levels. In this regard, a systematic review and meta-analysis of randomized, placebo-controlled clinical trials examining the effects of statins on leptin found that the current evidence remains inconclusive. These findings reinforce the idea that addressing obesity solely with the recommendation to “eat less and move more” is an oversimplification of a complex, multifactorial disease in which genetic, metabolic, hormonal, and environmental factors interact [44,45].

## 5. Conclusions

This study reinforces the understanding that the ghrelin–leptin hormonal axis plays a central role in the pathophysiology of obesity and in the metabolic response following metabolic and bariatric surgery. In particular, the influence of the type of surgical procedure on hormonal dynamics, leptin sensitivity, and metabolic parameters suggests that not all interventions have the same physiological impact, which could have relevant implications for personalized treatment selection. Therefore, these findings emphasize the need to continue exploring the molecular and hormonal mechanisms involved, including the role of the gut microbiome and inflammatory pathways, in order to optimize the clinical management of obesity and its comorbidities from a more comprehensive, precise, and personalized approach.

## 6. Limitations

There are important limitations to the present study, primarily its design as a nonrandomized, prospective cohort, which prevents the establishment of firm causal relationships between soluble Ob-R levels and adipose tissue function; we suggest a case–control design study in the future. Second, participants were treated with a variety of antihyperglycemic drugs, which could have influenced insulin levels and leptin or ghrelin secretory function, which could have affected the results related to the hormonal and metabolic parameters evaluated. Finally, the study population included patients with poor glycemic and lipidic control, as well as with other comorbidities such as gastrointestinal and emotional conditions and hypothyroidism which could affect the ghrelin–leptin axis.

## Figures and Tables

**Table 1:** Anthropometric, clinical, and biochemical characteristics in patients with severe obesity undergoing metabolic and bariatric surgery.

	TOTAL (n = 194)	RYBG (n = 36)	OAGB (n = 86)	SG (n = 72)
		Pre-Surgery	Pre-Surgery	Pre-Surgery
anthropometry				
Age (years)	40.6 ± 9.6	40.4 ± 10.7	41.4 ± 8.5	39.8 ± 10.2
Gender (M/F)	37/157	9/27	15/71	13/59
Weight Pre-Surgery (Kg)	114.9 ± 24.4	110.8 ± 26.8	117.2 ± 26.4	114.3 ± 20.8
BMI	43.1 ± 8.01	41.6 ± 6.9	44.7 ± 7.8	42.3 ± 8.9
WHR	0.97 ± 0.58	1.15 ± 1.29	0.9 ± 0.08	1 ± 0.1
Lean body mass (kg)	57.9 ± 11.4	58.2 ± 12.7	58.8 ± 11.1	56.7 ± 11.2
Body fat (kg)	55.6 ± 15.4	52.4 ± 17.3	57.4 ± 16.3	55 ± 13.5
Muscle mass (kg)	55.1 ± 10.5	56.2 ± 11.1	55.5 ± 10.3	54.3 ± 10.6
comorbidities				
T2DM (%)	37.6	63.8	33.7	29.1
HTN (%)	39.17	41.6	36	41.6
Dyslipidemia (%)	54.1	61.1	56.9	47.2
laboratory tests				
Leukocytes (×10^3^/mL)	8.1 ± 1.9	7.9 ± 1.8	8.5 ± 2.2	7.9 ± 1.7
Lymphocytes	3.2 ±5.5	3.9 ± 7.8	3.8 ± 7	2.2 ± 0.5
Hemoglobin (g/dL)	14.7 ±1.5	14.9 ± 0.9	14.8 ± 1.5	14.4 ± 1.7
Albumin (g/dL)	4.4 ± 5	6.7 ± 12.2	3.9 ± 0.3	3.9 ± 0.3
Hematocrit (%)	44.4 ± 6.3	45.2 ± 3	43.9 ± 8.3	44.5 ± 5.2
Platelets	275.1 ± 63.5	274.6 ± 48.6	273 ± 66.1	276.6 ± 67.5
Glucose (mg/dL)	106.5 ± 28.5	115.6 ± 42	107.3 ± 28.8	100.9 ± 15.9 *
HbA1c (%)	6.07 ± 1	6.7 ± 1.1	6.2 ± 1.3 *	5.6 ± 0.5 ***
Creatinine (mg/dL)	0.7 ± 0.17	0.7 ± 0.1	0.7 ± 0.1	0.7 ± 0.2
TC (mg/dL)	179.6 ± 34.5	176.5 ± 40.9	180.9 ± 34	179.8 ± 31.8
TG (mg/dL)	163.1 ± 77.2	168 ± 73.5	168.5 ± 88.7	154.7 ± 64.9
HDL-C (mg/dL)	40.2 ± 10	39.6 ± 10.4	40 ± 10	40.6 ± 9.8
LDL-C (mg/dL)	111.6 ± 30.2	105.4 ± 36.7	111.8 ± 30.6	114.4 ± 26.1

Roux-en-Y gastric bypass (RYBG), one anastomosis gastric bypass (OAGB), sleeve gastrectomy (SG). Data are expressed as n (%), mean ± standard deviation or percentage (%). M: male; F: female; BMI: body mass index; HbA1c: glycated hemoglobin; HTN: hypertension; HDL-C: high-density lipoproteins cholesterol; LDL-C: low-density lipoproteins cholesterol; TC: total cholesterol; TG: triglycerides; T2DM: type 2 diabetes mellitus; WHR: waist-to-hip ratio. * *p* < 0.05 RYBG vs. SG; *** *p* < 0.001 RYBG vs. SG.

**Table 2:** Pre-surgery body weight and percentage of excess weight loss (% EWL) after surgery.

TOTAL (n = 194)			RYBG (n = 36)		OAGB (n = 86)		SG (n = 72)	
	Kg	%EWL	Kg	%EWL	Kg	%EWL	Kg	%EWL
Pre-Surgery	114.9 ± 24.4	--	110.8 ± 26.8	--	117.2 ± 26.4	--	114.3 ± 20.8	--
Post-surgery times								
1 w	107.8 ± 24.3	27 ± 9.4	105 ± 27.2	27.2 ± 9.6	108.2 ± 24	27.4 ± 9.1	108.2 ± 23.5	26.6 ± 9.7
1 m	101.8 ± 25.8	35 ± 10.6	99 ± 27.6	37.7 ± 11.3	104.2 ± 24.2	33.6 ± 9.2	102 ± 24.8	35.4 ± 11.4
3 m	95.6 ± 22.2	47 ± 11.5	97.1 ± 29.9	50 ± 12.5	95.3 ± 21.7	47.7 ± 12.1	95.5 ± 19.5	45.2 ± 10.4
6 m	95.8 ± 72.3	56.9 ± 13.4	90.7 ± 24.9	56.5 ± 13.2	86.7 ± 19.5	59 ± 14.6	106 ± 107	55.3 ± 12.5
12 m	78 ± 17.9	65.2 ± 16	74.8 ± 8.6	47.1 ± 16.9	74.1 ± 19.4	65.6 ± 12.2	83.9 ± 16.8	64.8 ± 19.4

Roux-en-Y gastric bypass (RYBG), one anastomosis gastric bypass (OAGB), sleeve gastrectomy (SG). Mean ± SD (standard deviation). Pre-surgery: baseline; post-surgery times: 1 w: 1 week; 1 m: 1 month; 3 m: 3 months; 6 m: 6 months; 12 m: 12 months.

**Table 3:** Leptin, FLI, sOB-R, and ghrelin concentrations pre-surgery (baseline) and post-surgery.

	Leptin ANOVA		FLI ANOVA		sOB-R ANOVA		Ghrelin ANOVA	
	ng/mL	*p*-Value	ng/mL	*p*-Value	ng/mL	*p*-Value	ng/mL	*p*-Value
Pre-Surgery	25.9 ± 4	--	8.0 ± 3.9	--	4.6 ± 5	--	4.0 ± 2.5	--
post-surgery times								
1 w	25.5 ± 4.1	0.0250	9.5 ± 6.2	0.8644	3.8 ± 2	0.9975	1.6 ± 1.8 ^&^	<0.0001
1 m	23.7 ± 5.5	<0.0001	7.2 ± 3.3	0.9412	4.1 ± 2.5	0.9999	1.7 ± 2 ^&^	<0.0001
3 m	24.0 ± 5.5	<0.0001	7.1 ± 3.4	0.1694	5.1 ± 2.6	0.9997	2.2 ± 1.5	0.0009
6 m	22.7 ± 6.2	<0.0001	7.8 ± 4.9	0.9895	4.0 ± 2.9	0.9994	2.8 ± 2	0.0821
12 m	19.45 ± 3.4	<0.0001	5.1 ± 2.8	<0.0001	4.8 ± 3 ^&&&^	0.9867	3.1 ± 2.4	0.3211

Mean ± SD (standard deviation). ANOVA intragroup difference pre-surgery and post-surgery bariatric was *p* < 0.01. 1 m vs. 12 m (^&^ *p* < 0.05); 6 m vs. 12 m (^&&&^ <0.0001). FLI: free leptin index; sOB-R: soluble leptin receptor.

## Data Availability

The datasets used and/or analyzed during the current study are available from the corresponding author on reasonable request.

## Abbreviations

The following abbreviations are used in this manuscript:

BMI	Body mass index
BPD-DS	Biliopancreatic diversion with duodenal switch
CV	Cardiovascular disease
T2DM	Type 2 diabetes
ELISA	Enzyme-linked immunosorbent assay
ESR	Erythrocyte sedimentation rate
EWL	Excess weight loss
FLI	Free leptin index
HbA1c	Glycated hemoglobin A1c
HDL-C	High-density lipoprotein cholesterol
IR	Insulin resistance
LDL-C	Low-density lipoprotein cholesterol
MBS	Metabolic and bariatric surgery
OAGB	One anastomosis gastric bypass
RYGB	Roux-en-Y gastric bypass surgery
ROS	Reactive oxygen species
SG	Sleeve gastrectomy
sOB-R	Serum soluble leptin receptor
SOD	Superoxide dismutase
TC	Total cholesterol
TG	Triglycerides
TNF-α	Tumor necrosis factor alpha
VAT	Visceral adipose tissue
WAT	White adipose tissue
WHR	Waist-to-hip ratio
WMA	World Medical Association
